# Natural History of *Plasmodium odocoilei* Malaria Infection in Farmed White-Tailed Deer

**DOI:** 10.1128/mSphere.00067-18

**Published:** 2018-04-18

**Authors:** Ann M. Guggisberg, Katherine A. Sayler, Samantha M. Wisely, Audrey R. Odom John

**Affiliations:** a Department of Pediatrics, Washington University School of Medicine, St. Louis, Missouri, USA; b Department of Wildlife Ecology and Conservation, University of Florida, Gainesville, Florida, USA; c Department of Molecular Microbiology, Washington University School of Medicine, St. Louis, Missouri, USA; University at Buffalo

**Keywords:** malaria, *Odocoilei virginianus*, *Plasmodium*, *Plasmodium odocoilei*, veterinary parasitology, white-tailed deer

## Abstract

White-tailed deer (Odocoileus virginianus), an ecologically and economically important species, are the most widely distributed large animals in North America. A recent study indicated that up to 25% of all white-tailed deer may be infected with Plasmodium odocoilei, a malaria parasite belonging to the distinct clade of ungulate-infecting *Plasmodium* spp. Because the clinical impact of P. odocoilei on deer health and survival is unknown, we undertook a retrospective longitudinal study of farmed Floridian O. virginianus fawns. We found that a substantial proportion (21%) of fawns acquire malaria infection during the first 8 months of life. Some animals naturally clear P. odocoilei infection, while other animals remain persistently positive. Importantly, we found that animals that acquire malaria parasites very early in life have poor survival compared to animals that remain uninfected. Our report thus provides the first evidence of a clinically significant impact of malaria infection in young deer.

**IMPORTANCE** Malaria parasites of the genus *Plasmodium* are known to infect a variety of vertebrate hosts, including ungulates (hoofed mammals). A recent study found that up to a quarter of white-tailed deer (Odocoileus virginianus) in North America are infected with the parasite Plasmodium odocoilei. In addition to occupying an important ecological niche, white-tailed deer are popular game animals and deer farming represents a rapidly growing industry. However, the effect of P. odocoilei infection in this ecologically and economically important ungulate species is unknown. Our work is significant because (i) we identified a high prevalence of P. odocoilei in farmed deer and (ii) we found evidence for both cleared and persistent infection, as well as an association with decreased survival of young fawns.

## Abstract (precis)

Malaria parasites of the genus *Plasmodium* are known to infect a variety of vertebrate hosts, including ungulates (hoofed mammals). A recent study found that up to a quarter of white-tailed deer (Odocoileus virginianus) in North America are infected with the parasite Plasmodium odocoilei. In addition to occupying an important ecological niche, white-tailed deer are popular game animals and deer farming represents a rapidly growing industry. However, the effect of P. odocoilei infection in this ecologically and economically important ungulate species is unknown. Our work is significant because (i) we identified a high prevalence of P. odocoilei in farmed deer and (ii) we found evidence for both cleared and persistent infection, as well as an association with decreased survival of young fawns.

## INTRODUCTION

Wild ungulates comprise a diverse and highly successful group of hoofed mammals. Their close ecological and phylogenetic association with livestock such as cattle, goats, sheep, and pigs has made them a center of study for emerging infectious diseases at the livestock-wildlife interface for more than a century. More recently, ungulates have been prioritized in many One Health initiatives due in part to the close association of humans and domestic ungulates, which creates a conduit for zoonotic transmission (1). Nearly all domestic hoofstock host zoonotic pathogens (2), and approximately 32% of all wild ungulates host them as well (3). Ungulates are also hosts for a myriad of pathogens that cause vector-borne diseases such as babesiosis, trypanosomiasis, and Q fever (4), as well as directly transmitted diseases such as brucellosis, bovine tuberculosis, and multiple enteric pathogen syndromes (5).

Over the last century, scattered reports have indicated the presence of hemosporidian parasites in the blood of a variety of ungulate species, including duiker antelope, water buffalo, Asian mouse deer, and goats (6–9). Until recently, these studies were limited to morphological descriptions based on examination of blood smears of intraerythrocytic parasites that possess the characteristic features of *Plasmodium* species. In 2016, however, molecular characterization of blood-borne parasites of domestic water buffalo and African goats revealed that two ungulate malaria species, Plasmodium bubalis and Plasmodium caprae, are phylogenetically distinct from all other mammalian *Plasmodium* spp. and group within a single unique clade. Interestingly, the clade of ungulate-infecting *Plasmodium* spp. groups most closely with hemosporidian species that infect birds, bats, and lizards, rather than grouping with primate-infecting *Plasmodium* spp. Ungulate malarial species appear to have branched off after *Haemoproteus* and *Parahaemoproteus* spp. (which infect birds) and before *Polychromophilus* spp. (bats) and bird- and lizard-infecting *Plasmodium* spp. (9).

Malaria parasites also appear to be endemic in a geographically distant species of ungulate, the white-tailed deer (Odocoileus virginianus), the most widely distributed large animal in North America. An intraerythrocytic parasite consistent with malaria, described as Plasmodium odocoilei, had been morphologically described decades ago from blood smears of a splenectomized animal (10, 11). Surprisingly, a recent, broader survey of O. virginianus adults revealed that P. odocoilei is both common and widely distributed in North American white-tailed deer (12). P. odocoilei infection was confirmed in animals from eight U.S. states, with up to 25% of animals identified as infected at some sites. Phylogenetically, P. odocoilei groups together with P. bubalis and P. caprae within the distinct clade of ungulate malaria parasites (13). While the manuscript was under review, a new study also identified *Plasmodium* parasites infecting Pampas deer (Ozotoceros bezoarticus) in South America. Sequences from these parasites also fall within the clade of ungulate-infecting *Plasmodium* and group most closely with P. odocoilei (14).

Clinical characterization of ungulate malaria infection has been limited. For all species examined (P. odocoilei, P. bubalis, and P. cephalophi), parasites have not been readily detected microscopically upon examination of blood smears from supposedly infected (nucleic acid-positive) animals (9, 12). The presumably low blood parasite burden observed in these adult individuals has led to the hypothesis that blood-stage ungulate malaria, especially that caused by P. odocoilei infection of O. virginianus, is best characterized as a chronic, occult infection without major health consequences (13).

For many human infections, adult and pediatric patients may have markedly different clinical presentations and health outcomes. For example, infection with respiratory syncytial virus (RSV) most often leads to minor upper respiratory illness in adults but represents a leading cause of hospitalization of and life-threatening respiratory distress in young infants (15). This distinction is also present in human P. falciparum malaria infection, as semi-immune adults may have low-level, “submicroscopic” parasitemia that can persist asymptomatically for extended periods of time, a particularly common characteristic in regions with high transmission intensity (16). However, in young infants and children, P. falciparum infection may result in characteristic and severe clinical syndromes, such as pronounced anemia and cerebral malaria (17). Given the discrepant clinical outcomes of many different human infections between pediatric and adult patients, we undertook a pilot study to determine whether P. odocoilei infection was present in young farmed Floridian fawns and to establish the clinical consequences of deer malaria in immature and immunologically naive animals.

## RESULTS

Information on the natural course of P. odocoilei infection was obtained through a longitudinal study of farmed O. virginianus fawns in the Panhandle of Florida. In brief, this study was performed in cooperation with the University of Florida (UF) Cervidae Health Research Initiative (CHeRI), which facilitates private industry-academic institution partnerships for deer health research. This pilot study was a retrospective cohort study, taking advantage of blood samples and survival data previously acquired for the study of epizootic hemorrhagic disease virus (EHDV) epidemiology. Blood was sampled from enrolled fawns at 3, 6, and 8 months of life. In addition, outcomes, including survival and release, were recorded for each animal.

We found that a substantial proportion (7/33 fawns = 21%) of O. virginianus fawns had acquired P. odocoilei infection at least once during the first 8 months of life (Table 1). At any given time point, more than 10% of all fawns examined were infected with P. odocoilei. Of the seven serially sampled fawns that acquired P. odocoilei infection during this study, four were *Plasmodium* blood PCR positive at the initial time point (3 months of life [September]), while two additional animals were first positive at 6 months of life (January), and a final animal was positive for the first time at 8 months of life (April). Our study results thus indicate that infection with malaria parasites is common in farmed white-tailed deer.

**TABLE 1:** P. odocoilei-positive, serially sampled O. virginianus fawns

Animal	Result^a^		
	3 mos	6 mos	8 mos
OV136	N	N	Y
OV137	N	Y	Y
OV115	Y	Y	Y
OV095	Y	N/A	N/A
OV141	Y	N/A	N/A
OV051	N	Y	N
OV035	Y	N	N

a Y, blood PCR positive for P. odocoilei; N, blood PCR negative (i.e., the result was below the limit of detection); N/A, sample unavailable due to death or release of fawn.

Based on sequence analysis of all P. odocoilei *cytB* amplicons obtained through this study, we found that there are multiple P. odocoilei strains circulating within the Florida sample population. This finding is consistent with the previous study of P. odocoilei sampled in the eastern United States, which identified six different *cytB* alleles (12). Many of our strains have *cytB* genotypes identical to those of previously identified P. odocoilei sequences. In our population, we identified strains with all but one of the previously described *cytB* alleles, including the divergent “allele A,” hypothesized by Martinsen et al. to originate from a distinct species of *Plasmodium* (Table 2) (12). We also identified a novel *cytB* allele (“allele D”) in the 8-month sample of OV136. As our primer sets were unbiased with respect to *Plasmodium* spp., our results support previous observations that white-tailed deer appear to be infected only with multiple closely related P. odocoilei strains, because no other *Plasmodium* spp. were detected.

**TABLE 2:** P. odocoilei *cytB* alleles identified in this study and in a previous study^a^

*cytB* allele	Sample(s) with identified allele	
	This study	Study by Martinsen et al. [12])
Allele A	OV115_8mo	APUN01
	OV137_8mo	APUN02
	OV137_6mo	
	OV141_3mo	
		
Allele B	None	ODVIR04
		
Allele C	OV035_3mo	ODVIR03
	OV095_3mo	
	OV115_6mo	
		
Allele D	OV136_8mo	None reported
		
Allele E/F	OV051_6mo*	ODVIR01 (E)
	OV115_3mo*	ODVIR02 (E)
		ODVIR06 (E)
		ODVIR05 (F)

a P. odocoilei *cytB* alleles were also identified in a previous study by Martinsen et al. (12). Samples that grouped together had 100% identity over the available *cytB* sequence. We identified a novel *cytB* allele (“Allele D”) in the OV136 sample. For the samples indicated with an asterisk (*), sequences covering the sites used to distinguish between alleles E and F were not available. The partial *cytB* sequences that we obtained during this study have been deposited in GenBank (accession no. MG709243 to MG709252).

In the two fawns that had sequential positive samples (OV137 and OV115), different *cytB* genetic signatures were detected. For OV137, the 6- and 8-month samples were genetically identical over the 171-bp *cytB* sequence that was available for all samples, as well as over a longer amplicon (574 bp) that was common to both the 6- and 8-month OV137 samples. This is suggestive of a single continuous infection, although the data are not conclusive. In contrast, for OV115, the *cytB* sequences from each time point appeared to be different. While these *cytB* alleles are distinct from each other, they match *cytB* alleles found in other, independent infections (Table 2). However, the OV115 6-month *cytB* sequence showed two sites with double peaks on the Sanger chromatogram, highly suggesting the presence of a mixed infection. The minor peaks, in combination with the rest of the *cytB* sequence from the 6-month samples, represent novel P. odocoilei *cytB* alleles and suggest the presence of an additional, cryptic strain of P. odocoilei. Future collection and sequencing of single infections with these alleles will help resolve the *cytB* haplotype(s) present in the population.

We observed a range of outcomes of *Plasmodium* infection in O. virginianus. Because symptoms associated with disease were not recorded, possible outcomes are limited to resolution of infection, persistence of infection, or death. Two infected animals had subsequent samples in which P. odocoilei nucleic acid was not detected, suggesting that these animals either had naturally resolved the infection or were capable of controlling parasite replication at levels below the limit of detection. In contrast, two animals were repeatedly positive over a period of several months, suggesting either that P. odocoilei is capable of persistent parasitemia or that there was sufficient infection pressure for animals to have become reinfected during the course of study.

**FIG 1:**
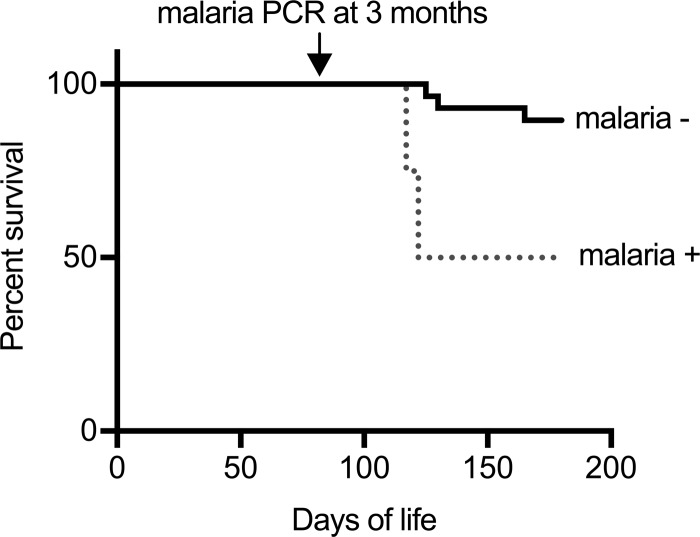
Plasmodium odocoilei infection status at 3 months predicts early O. virginianus survival. A Kaplan-Meier plot indicates the trend toward increased risk of mortality in fawns infected with P. odocoilei at 3 months of life compared to uninfected fawns within the same herd (*t* test [n.s., *P* = 0.07]).

Importantly, we examined whether P. odocoilei infection in O. virginianus is associated with increased mortality. In humans living in locations of malaria endemicity, malaria infection is most life-threatening during early childhood, with the majority of attributable deaths occurring in children under the age of 5, prior to the acquisition of immunity (17). Similarly, we found that P. odocoilei infection in the youngest animals was associated with a pronounced trend toward increased all-cause mortality by 6 months (Fig. 1), with 7% (2/27) mortality among malaria-negative animals and 50% (2/4) mortality among malaria-positive animals (odds ratio [OR], 12; *P* = 0.07).

**FIG 2:**
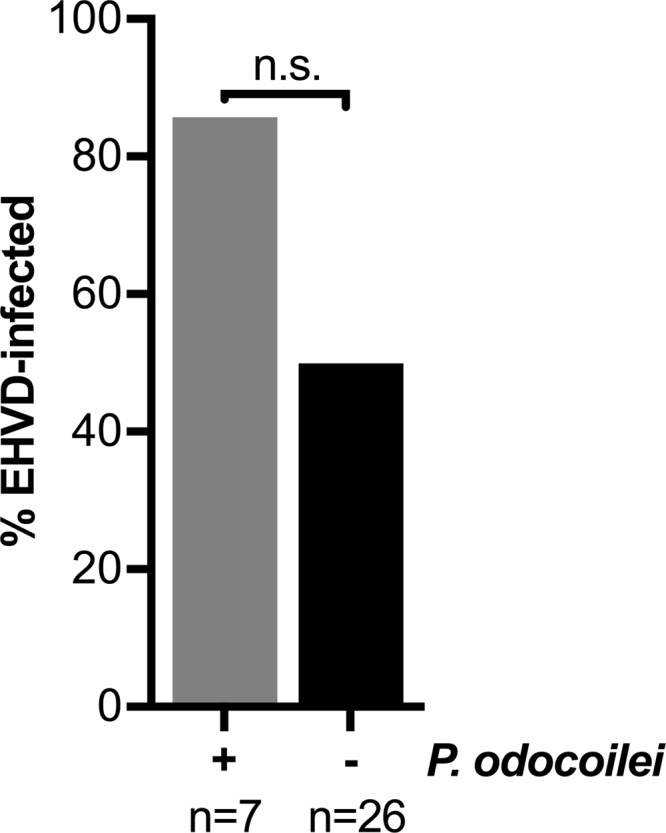
Possible association with P. odocoilei infection and life-threatening viral coinfection. Data represent proportions of animals infected with EHDV at any time point among the fawns with or without P. odocoilei infection (*t* test [n.s., *P* = 0.195]).

The cause of death in animals testing positive for P. odocoilei was not definitively determined. However, malaria in humans has a substantial impact on the risk for and outcome of coinfections (18–21). For this reason, we evaluated the prevalence of a life-threatening infectious virus (epizootic hemorrhagic disease virus [EHDV]) in fawns with or without *Plasmodium* infection. We found that a higher percentage of *Plasmodium*-infected fawns than of *Plasmodium*-negative controls was EHDV coinfected (Fig. 2) (86% [6/7] versus 50% [13/26] of controls; *P* = 0.125). This finding suggests that *Plasmodium* infection may be associated with an increased risk of acquiring EHDV or may alter viral control. Furthermore, the acute clinical decompensation and presence of EHD viral RNA in heart, spleen, and lung tissues at necropsy strongly suggest that EHDV contributed to death in *Plasmodium*-infected fawns.

## DISCUSSION

In this work, we provide the first longitudinal natural history study of naturally acquired nonprimate mammalian malaria parasites. We establish that P. odocoilei is highly prevalent in this population of Florida white-tailed deer fawns, with nearly one-quarter of all animals acquiring malaria parasites at some point during the first year of life. Importantly, we found that malaria infection is associated with poor clinical outcome, as young fawns infected with malaria parasites by 3 months of life are less likely to survive than uninfected age-matched peers in the same herd. Thus, our study results suggest that ungulate malaria may exert a substantial negative health impact, even if infection of adult animals leads to low parasite burdens and does not generally cause overt symptoms.

We found that P. odocoilei infection can occur in very young animals, with a substantial burden of P. odocoilei at the earliest time point in this study (3 months of life [September]), coincident with the probable timing of waning maternal antibody titers (22, 23). Because neonatal samples were unavailable, our study data cannot be used to distinguish vertical (doe-to-fawn) parasite transmission from mosquito-borne parasite transmission in the youngest animals. In humans, maternal antibodies are semiprotective and 95% of all symptomatic malaria in human children therefore occurs after 6 months of life (24), as maternal immunoglobulin levels fall. Earlier samples will be required to define the age of acquisition of first-time P. odocoilei infection in O. virginianus and whether congenital malaria infections, which are rare in humans, may be present.

The frequency of new infections and overall prevalence of malaria in our study population together suggest that there is intense transmission pressure for P. odocoilei. This idea is well supported by the existing entomological evidence, which indicates high rates of parasite infection in the possible mosquito vector species Anopheles punctipennis. The sole molecular survey of A. punctipennis, during which P. odocoilei was unexpectedly identified, noted a 5.7% vector infection rate in the survey location (Washington, DC), although careful entomological studies have yet to be performed to confirm the vector competence of A. punctipennis (12). However, for comparison, this is indicative of a higher rate of vector positivity than was recently observed in broad surveys of *Anopheles* spp. across Africa, during which a mean P. falciparum infection rate (measured by circumsporozoite protein enzyme-linked immunosorbent assay [CSP-ELISA]) of 3.37% (95% confidence interval [CI], 3.13 to 3.60) was found (25). The entomological inoculation rate (EIR), defined as the number of infectious bites per animal per year, is widely considered the “gold standard” metric to express the intensity of malaria parasite transmission under field conditions (26). Precise calculation of the EIR for P. odocoilei in Florida will rely on future studies of local mosquito sampling and calculation of the (likely substantial) mosquito biting rate for O. virginianus, but existing evidence suggests the EIR is comparable to that of human malaria in sub-Saharan Africa. Intriguingly, several deer species possess globin polymorphisms that cause erythrocyte sickling, and recent evolutionary studies suggest that these genetic changes may be undergoing positive selection (27). Human sickle cell heterozygosity confers protection against severe malaria (28). Because malaria parasite transmission pressure may be intense for deer, and because our studies suggest that infection in young white-tailed deer may impact survival, the possible association between deer erythrocyte sickling and malaria-related mortality is of great interest.

Our study results also indicate a high frequency of EHDV coinfection in fawns with P. odocoilei malaria, although this association was not statistically significant in our small pilot study. EHD is a vector-borne disease, transmitted by biting midges (*Culicoides* spp.) (29), many of which thrive under the moist conditions also preferred by the *Anopheles* spp. that typically transmit *Plasmodium* spp. For this reason, frequent coinfections may reflect common habitat-related exposures in individual fawns. However, *Plasmodium*-EHDV coinfections were often fatal in our cohort. Poor control of viral coinfections, including coinfections with HIV and herpesviruses, is a well-recognized complication of human malaria due to *Plasmodium* infection (18, 21). *Plasmodium*-susceptible ungulate species, including white-tailed deer and water buffalo, are likewise susceptible to a variety of host-restricted pathogens (foot-and-mouth disease virus and herpesvirus) (30, 31) and zoonotic viral pathogens (West Nile virus, Rift Valley fever viruses, and other arboviruses) (32–35). Therefore, future studies to understand the interplay between *Plasmodium* infection and control of viral infections in ungulates are needed due to the potential ecological, agricultural, and public health impacts of these coinfections.

Many knowledge gaps remain about ungulate malaria parasites in general and about P. odocoilei in particular. Key limitations of our study included its small sample size and relatively sparse sampling frequency. Current understanding of ungulate malaria biology is also significantly hampered by the lack of genomic information. The P. odocoilei genome has not been characterized; neither has that of any other representative ungulate malaria parasite species. As such, all aspects of its molecular genetics and gene repertoire are unknown, which hampers serological studies of prevalence and molecular epidemiological investigations. Of particular interest will also be the multigene families (such as the family of *var* genes) that mediate adhesion, virulence, and immune evasion in other *Plasmodium* spp. Comparative genomics has also already revealed unexpected and fundamental differences between murine and primate malaria parasite species in metabolic adaptations (36). Characterization of a divergent ungulate-infecting relative, such as P. odocoilei, will likely inform the fundamental adaptations required to adapt to the mammalian erythrocyte niche. To advance our fundamental understanding of the biology of this parasite, establishment and annotation of a complete reference genome are high priorities.

## MATERIALS AND METHODS

### Field site.

All studies were performed under the approval of the University of Florida IACUC (IACUC Study 201609390). Fawns were enrolled from a privately owned farm in Gadsden County, FL, that is permitted by Florida Department of Agriculture and Florida Fish and Wildlife Commission to keep, breed, and sell white-tailed deer (Odocoileus virginianus). All live-born fawns surviving to 3 months of age were enrolled for further study. Diagnostic blood samples were obtained from client-owned, farmed O. virginianus fawns (*n* = 33), enclosed within a single 500-acre preserve, at 3, 6, and 8 months of life. The outcomes for all study participants, including death, release (i.e., farmers may release healthy fawns to a preserve), and continued enclosure, were recorded.

### Sample collection.

Deer were held within approximately 1-acre high-fence enclosures of improved pastures seeded with Florida native grasses and Bahia grass. From each animal, 10 ml of whole blood was collected by jugular venipuncture during routine handling while animals were briefly mechanically immobilized using a squeeze chute designed for deer. All animal handling was approved by the UF Animal Care and Use Committee. To minimize handling episodes, animals were handled at 3 months of age (concordant with vaccine administration and weaning from does) and at 6 months (to sort individuals by sex) and at 8 months of age (concordant with vaccine booster administration). Blood was stored in EDTA at −4°C for no more than 12 h, prior to frozen storage at −80°C. The samples used in this study were archived and have not yet been published. Giemsa staining of blood smears was not available for these samples.

### Nucleic acid detection.

At each time point, whole-blood DNA was extracted from a 200-μl volume of whole blood using a commercial kit (DNeasy blood and tissue kit; Qiagen, Valencia, CA) according to the manufacturer’s instructions. Extracted DNA was evaluated by nested PCR for Plasmodium odocoilei analyses and by quantitative PCR (qPCR) for epizootic hemorrhage disease virus (EHDV) analyses. For screening of EHDV, total RNA was extracted using a standard magnetic bead protocol (Kingfisher Duo Prime; Thermo Fisher Scientific). Multiplex quantitative reverse transcription-PCR (qRT-PCR) (37) was used to determine the presence of EHDV or Bluetongue virus (BTV) viral RNA. Screening for P. odocoileus was performed via nested PCR using pan-*Plasmodium* primers, as previously described (12, 38). Briefly, primers amplifying a portion of the *cytB* locus (12, 39) were used in a PCR mixture containing a 2 µM concentration of each primer, 2 to 50 ng total DNA, and Kapa HiFi HotStart PCR mix. A 2-μl volume of the reaction mixture was used as a template for the nested PCR. Optimal primer annealing temperatures were determined under the described conditions, and the extension temperature for all PCRs was reduced to 68°C to accommodate AT-rich templates. Due to high sensitivity, nested PCRs were performed in an otherwise *Plasmodium*-free laboratory to reduce the possibility of false positives due to trace DNA. Additionally, a negative “no-template” control consisting of molecular-analysis-grade water was included for each set of PCRs. All nested PCR amplicons were additionally sequenced via Sanger sequencing and compared to P. odocoilei *cytB* sequences (12). Using serial dilutions of plasmid DNA encoding a *Plasmodium cytB* sequence, the limit of detection of our nested PCR strategy was determined to be ~0.2 *cytB* copies per reaction.

### Accession numbers.

Nucleic acid sequences of the partial *cytB* coding sequences (CDS) from samples described in this study have been deposited in GenBank under the following accession numbers: MG709243, MG709244, MG709245, MG709246, MG709247, MG709248, MG709249, MG709250, MG709251, and MG709252.
